# Supplementary material for: Studies of mice with a large deletion of the ARPKD-associated Pkhd1 locus likely explain its GWAS association with glaucoma in humans
Source: bioRxiv. 2026 Feb 17:2026.02.15.706040. Preprint. [Version 1] doi: 10.64898/2026.02.15.706040 (PMC12934734; doi:10.64898/2026.02.15.706040)
Supplement: Supplement 1 [file NIHPP2026.02.15.706040v1-supplement-1.pdf]

Supplementary Fig.1: Characterization of the eye phenotype of *Pkhd1*$^{del3\text{-}67/del3\text{-}67}$ mutants.

bioRxiv preprint doi: https://doi.org/10.64898/2026.02.15.706040; this version posted February 17, 2026. The copyright holder for this preprint (which was not certified by peer review) is the author/funder. This article is a US Government work. It is not subject to copyright under 17 USC 105 and is also made available for use under a CC0 license.

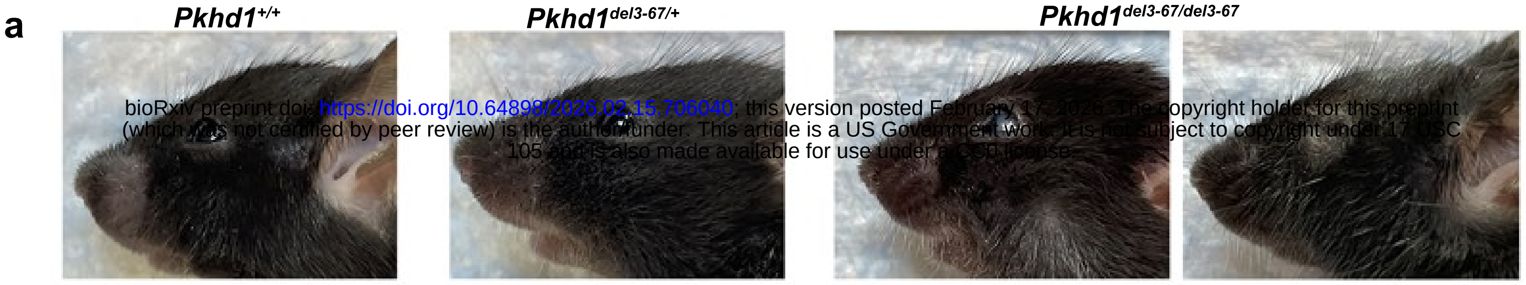

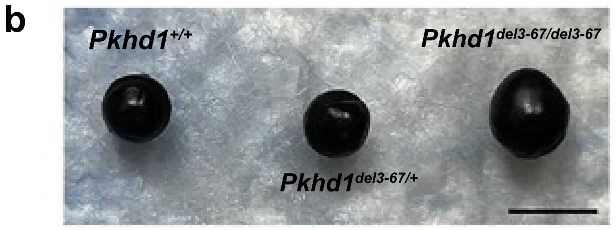

c

Pkhd1 Genotype	Number of mice	Eye phenotype (%)
WT	203	0 / 203 = 0 %
Pkhd1 $^{del3\text{-}67/+}$	484	0 / 484 = 0 %
Pkhd1 $^{del3\text{-}67/del3\text{-}67}$	178	178 / 178 = 100 %

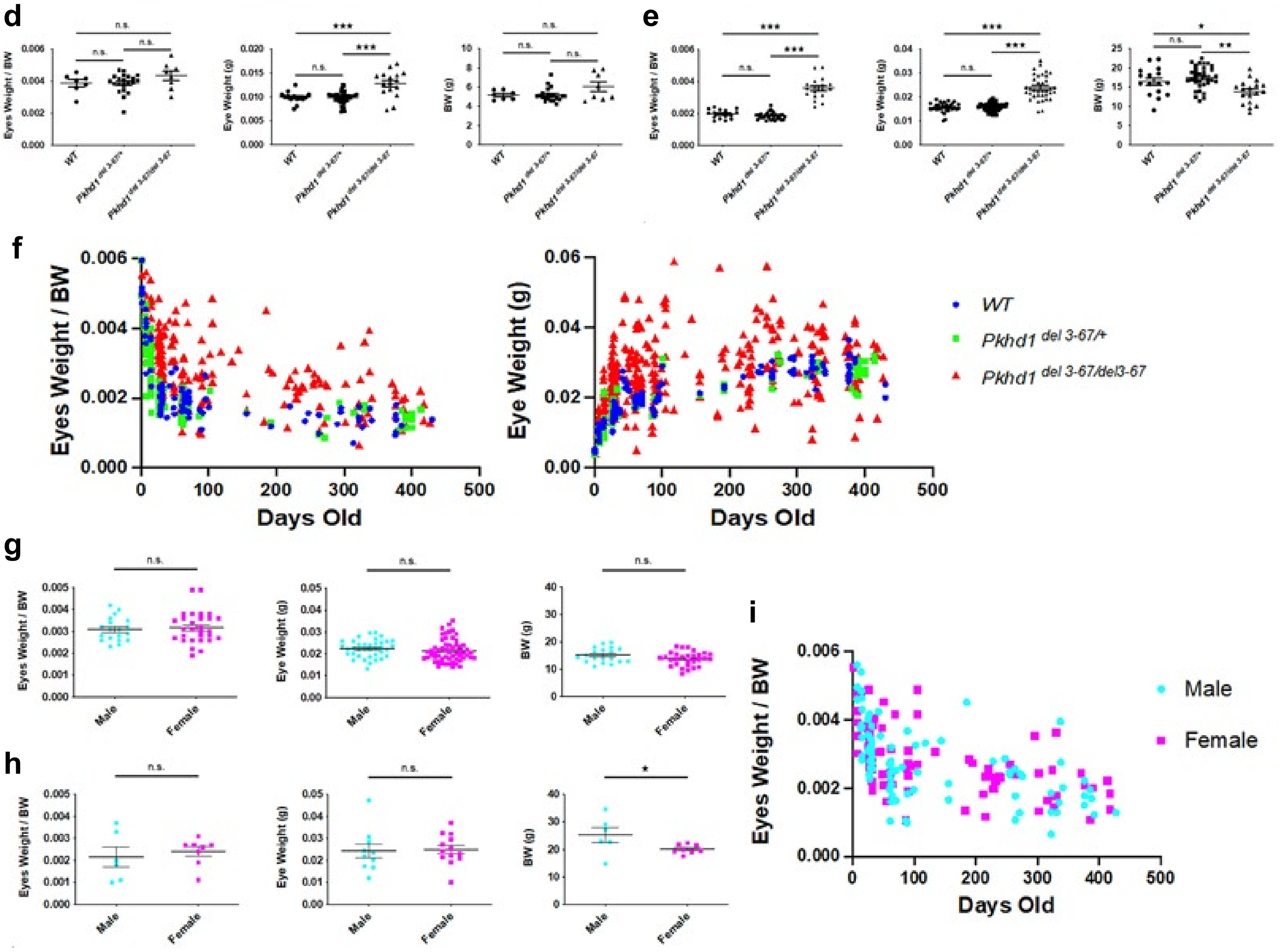

(a) Representative images of eyes from *Pkhd1*$^{del3\text{-}67/del3\text{-}67}$ and littermate controls at 2 months of age. All *Pkhd1*$^{del3\text{-}67/del3\text{-}67}$ mice developed corneal opacity but as shown in the right some of the mice develop atrophic recessed eyes with scarred eyelids as they age. (b) Representative eye globes of *Pkhd1*$^{del3\text{-}67}$ mouse line at 3 months of age. All mice are littermates. (c) Prevalence of eye phenotype in the *Pkhd1*$^{del3\text{-}67}$ mouse line. (d) From left, eyes weight per body weight, eye weight and BW at 1 week of age. For Supplementary Figure 1d-i, each dot in Eyes Weight/BW is the mean value of eyes from a single mouse while each dot in eye weight indicates the measurement for a single eye.

in Eye Weight indicates a single eye. ★★★ $p < 0.001$, ★★ $p < 0.01$, ★ $p<0.05$. n.s. not significant).

(f) Eye size at different ages of *Pkhd1*$^{del3\text{-}67}$ mouse line. left; scatter plots of eyes weight per BW and right; eye weight at different ages.

(g) Comparison of eye size of *Pkhd1*$^{del3\text{-}67del3\text{-}67}$ male and female mice at around 1 month of age. From left: eyes weight per BW, eye weight and BW. There are no significant differences in these parameters.

(h) Comparison of eye size of *Pkhd1*$^{del3\text{-}67del3\text{-}67}$ male and female mice at around 3 months of age. From left: eyes weight per BW, eye weight and BW. Mice are 90 ± 3 days old mice. ★ $P < 0.05$. n.s. not significant.

(i) Scatter plot of eyes per BW of Pkhd1del3-67del3-67 of male and female mice. There is no evidence to suggest there is a significant difference in eye phenotype of *Pkhd1*$^{del3\text{-}67del3\text{-}67}$ based on sex.

Supplementary Fig.2: Additional data for *Pkhd1$^{del3-67/del3-67}$* mouse eye pathology.

bioRxiv preprint doi: https://doi.org/10.64898/2026.02.15.706040; this version posted February 17, 2026. The copyright holder for this preprint (which was not certified by peer review) is the author/funder. This article is a US Government work. It is not subject to copyright under 17 USC 105 and is also made available for use under a CC0 license.

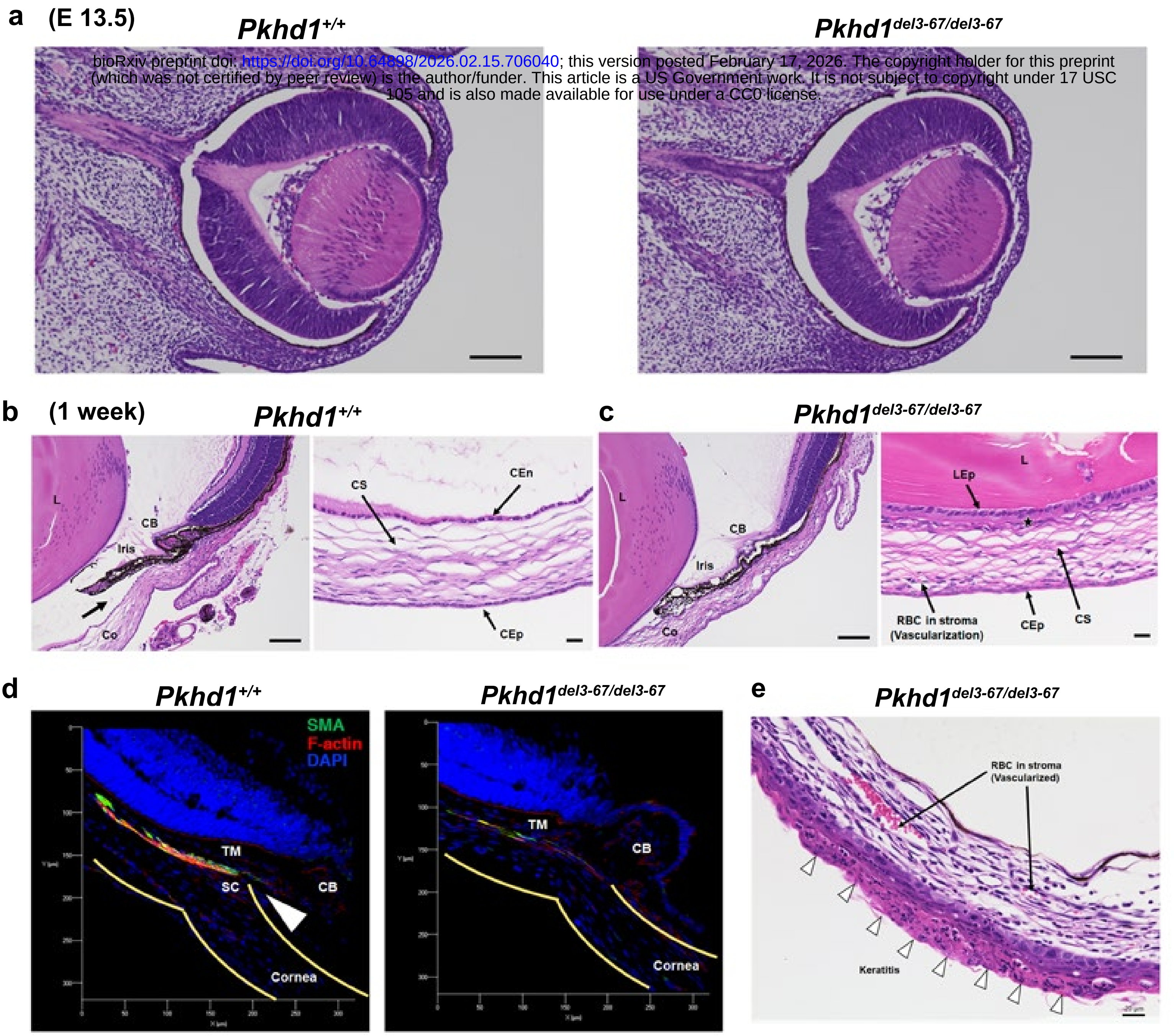

(a) Representative H&E staining pattern of E13.5 control (left) and littermate (right) *Pkhd1$^{del3-67/del3-67}$* eyes showing no differences. Scale bar 100 µm.

(b, c) Representative eye pathology at 1 week of age (H&E staining). Left: angle tissue with 100 µm scale bar; Right: cornea with 20 µm scale bar. (b) Littermate control. The arrow in the left panel identifies the open angle in the control which is absent in the mutant. (c) *Pkhd1$^{del3-67/del3-67}$* mutants also have abnormal ciliary body, lack a corneal endothelium, and have a vascularized corneal stroma. CB: ciliary body, Co: cornea, CEn: corneal endothelium, CS: corneal stroma, CEp: corneal epithelium, L: lens, LEp: lens epithelium. The star indicates absent corneal endothelium in *Pkhd1$^{del3-67/del3-67}$*.

(d) Immunofluorescence of the angle of the anterior chamber at 4 weeks of age. Left: control, right: *Pkhd1$^{del3-67/del3-67}$*. The arrowhead indicates the angle of the anterior chamber, which is closed in *Pkhd1$^{del3-67/del3-67}$*. TM: trabecular meshwork, SC: Schlemm's canal, CB: ciliary body. Yellow line indicates surface of the eye and cornea. Scale is shown in µm.

(f) 1 month old *Pkhd1$^{del3-67/del3-67}$* eye with keratitis and obvious vascularization. Arrow heads indicate keratitis.

Supplementary Fig.3: Evaluation of retinal layers of *Pkhd1*$^{del3\text{-}67/del3\text{-}67}$.

bioRxiv preprint doi: https://doi.org/10.64898/2026.02.15.706040; this version posted February 17, 2026. The copyright holder for this preprint (which was not certified by peer review) is the author/funder. This article is a US Government work. It is not subject to copyright under 17 USC 105 and is also made available for use under a CC0 license.

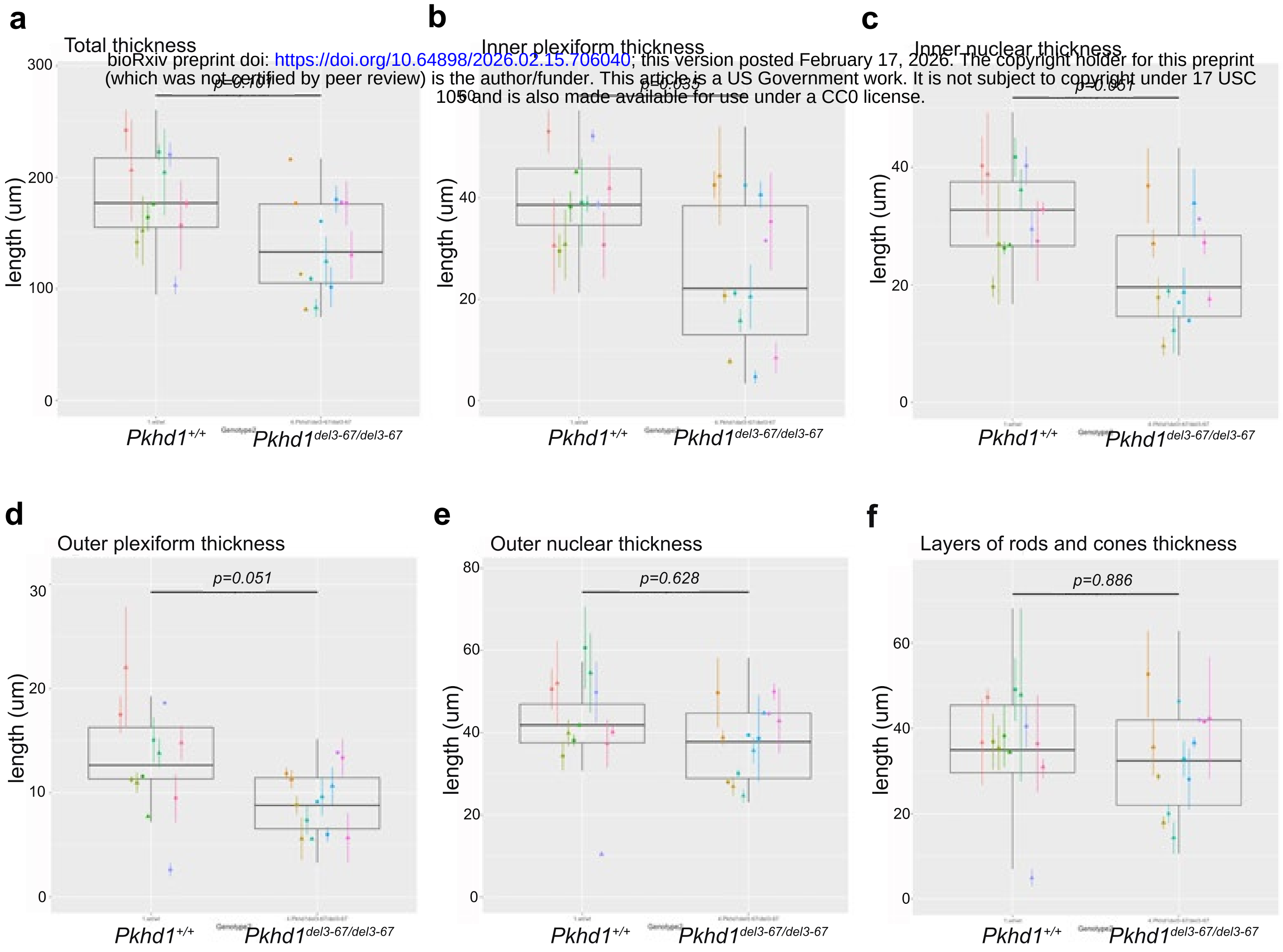

Box plot showing thickness of layers of retina in *Pkhd1*$^{del3\text{-}67/del3\text{-}67}$ (n=7) and *Pkhd1*$^{+/+}$ (n=6) 1-month-old mice.
(a) Total retina, (b) inner plexiform layer, (c) inner nuclear layer, (d) outer plexiform layer, (e) outer nuclear layer, (f) rods and cones. Each dot is the average thickness and the range indicates maximum and minimum values per eye; the left and right eye of a single mouse are colored the same. The bars show Wilcoxon rank sum p-values.

Supplementary Fig.4: Transcriptomic analysis of human and mouse eye.

bioRxiv preprint doi: https://doi.org/10.64898/2026.02.15.706040; this version posted February 17, 2026. The copyright holder for this preprint (which was not certified by peer review) is the author/funder. This article is a US Government work. It is not subject to copyright under 17 USC 105 and is also made available for use under a CC0 license.

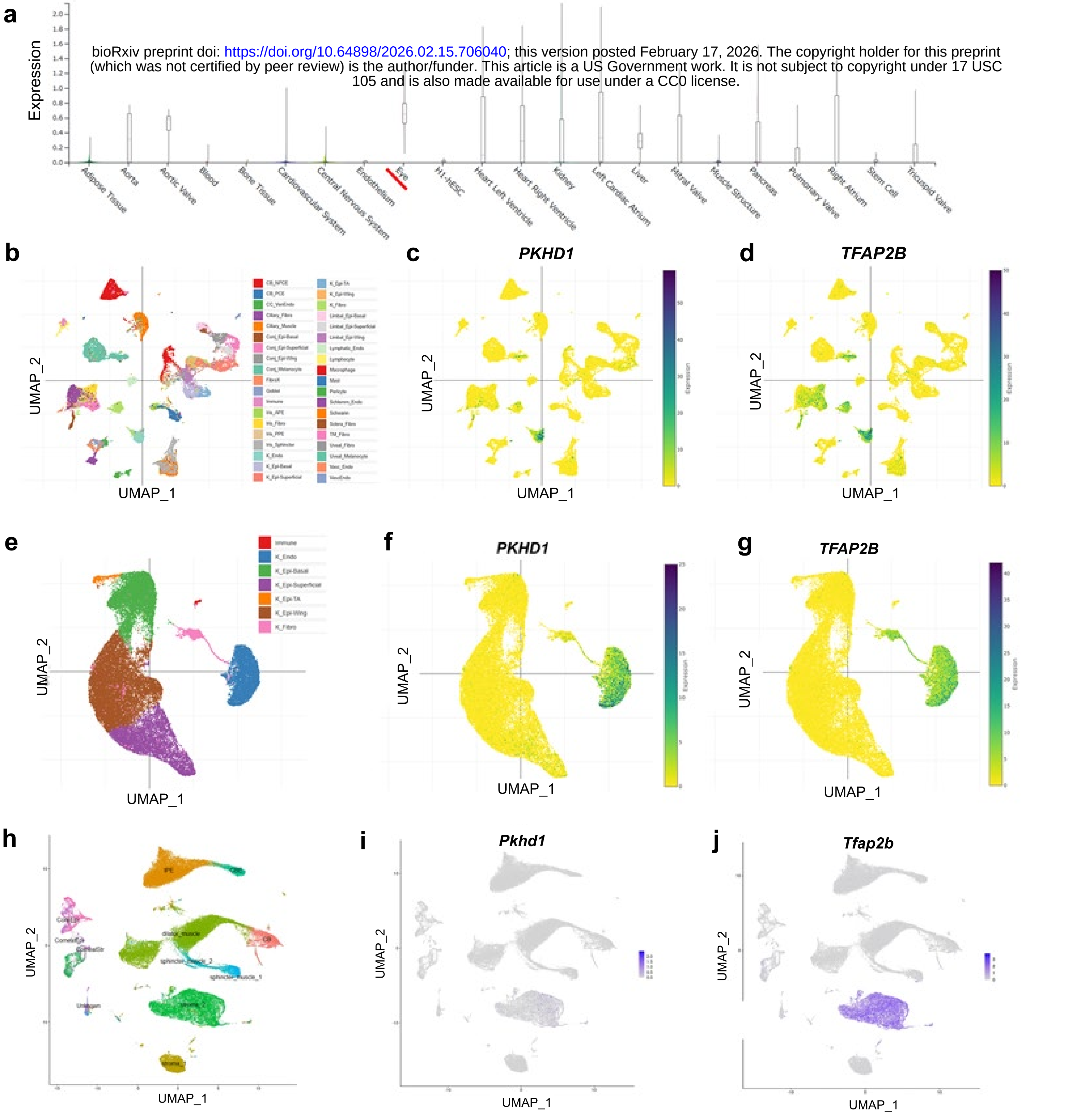

(a) Tissue-specific *PKHD1* transcripts expression from the Common Metabolic Diseases Knowledge Portal. Median *PKHD1* expression level is highest in the eye.
(b-d) UMAP plots of adult human eye data of integrated cell type clusters. (b) clusters included all cell types; CB_NPCE: ciliary body nonpigmented ciliary epithelium, CB_PCE: ciliary body pigmented ciliary epithelium, CC_VenEndo: collector channel/venous endothelium, Ciliary Fibro: ciliary fibroblast, Conj_Epi_Basal: conjunctival epithelium basal, Conj_Epi_Superficial: conjunctival epithelium superficial, Conj_Epi_Wing: conjunctival epithelium wing, Conj_Melanocyte: conjunctival melanocyte, FibroX: transcriptomically similar to scleral fibroblast but exhibit distinct markers, Goblet: goblet cell, Immune: immune cell, Iris_APE: iris anterior pigmented epithelium, Iris_Fibro: iris fibroblast, Iris PPE iris posterior pigmented epithelium:, K_Endo: corneal endothelium, K_Epi_Basal: corneal epithelium basal, K_Epi_Superficial: corneal epithelium superficial, K_Epi_TA: corneal epithelium transit amplifying, K_Epi_Wing: corneal epithelium wing, K_Fibro: corneal fibroblast, Limbal_Epi_Basal: limbal epithelium basal, Limbal_Epi_Superficial: limbal epithelium superficial, Limbal_Epi_Wing: limbal epithelium wing, Lymphatic_Endo: lympatic endothelial, Mast: mast cell, Schlemm_Endo: Schlemm canal endothelium, Schwann: Schwann cell, Sclera_Fibro: sclera fibroblast, TM_Fibro: trabecular meshwork fibroblast, Uveal_Fibro: uveal fibroblast, Uveal_Melanocyte: uveal melanocyte, Vasc_Endo: vascular endothelium, VascEndo: transcriptomically similar to vascular endothelium but exhibit distinct markers.
(c) *PKHD1* expression pattern in all clusters. *PKHD1* expression is mostly in the "corneal endothelial cell" cluster (dark: higher expression); (d) *TFAP2B* expression in all clusters. *TFAP2B* expression is highest in corneal endothelial cells, fibroblasts, and conjunctival melanocytes.
(e-g) UMAP plots of adult human eye data restricted to cell types present in the corneal sub-cluster. This figure is derived from the same dataset used in Supplementary Figure 4b-d, which included all cell types. Cell types present in the corneal sub-cluster: Immune: immune cell, K_Endo: corneal endothelium, K_Epi_Basal: corneal epithelium basal, K_Epi_Superficial: corneal epithelium superficial, K_Epi_TA: corneal epithelium transit amplifying, K_Epi_Wing: corneal epithelium wing, K_Fibro: corneal fibroblast.
(h-j) UMAP plots of adult mouse eye data showing (h) cell type clusters, *Pkhd1* (i), and *Tfap2b* expression (j).

Supplementary Fig.5: *Pkhd1* exon read number and potential enhancer region.

a ***Pkhd1* exon Number**

bioRxiv preprint doi: https://doi.org/10.64898/2026.02.15.706040; this version posted February 17, 2026. The copyright holder for this preprint (which was not certified by peer review) is the author/funder. This article is a US Government work. It is not subject to copyright under 17 USC 105 and is also made available for use under a CC0 license.

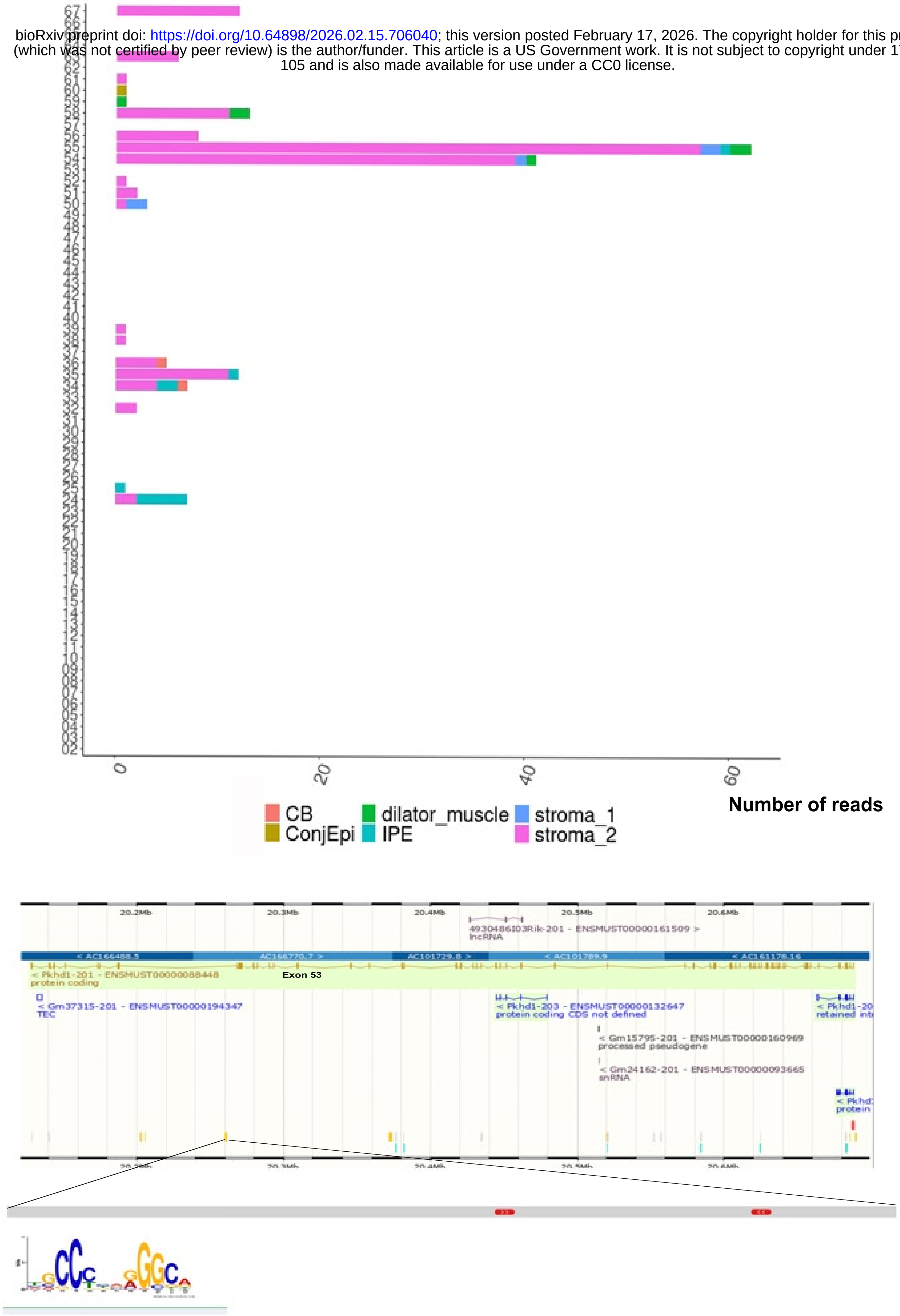

(a) Number of reads found for each *Pkhd1* exon in select eye cell populations, colored by cell type. Note the high number of reads in genomic exons 54 and 55, particularly in stroma 2 cells. IPE: iris pigmented epithelium, CB: ciliary body, ConjEpi: conjunctival epithelium.
(b) UCSC Genome Browser map of *Pkhd1* genomic region showing enhancers in yellow. An enhancer in the vicinity of exons 54 and 55 is enlarged to show the location of AP-2β motifs

Supplementary Fig.6: RNA *in situ* hybridization for *Pkhd1* transcripts in the eye.

https://doi.org/10.64898/2026.02.15.706040

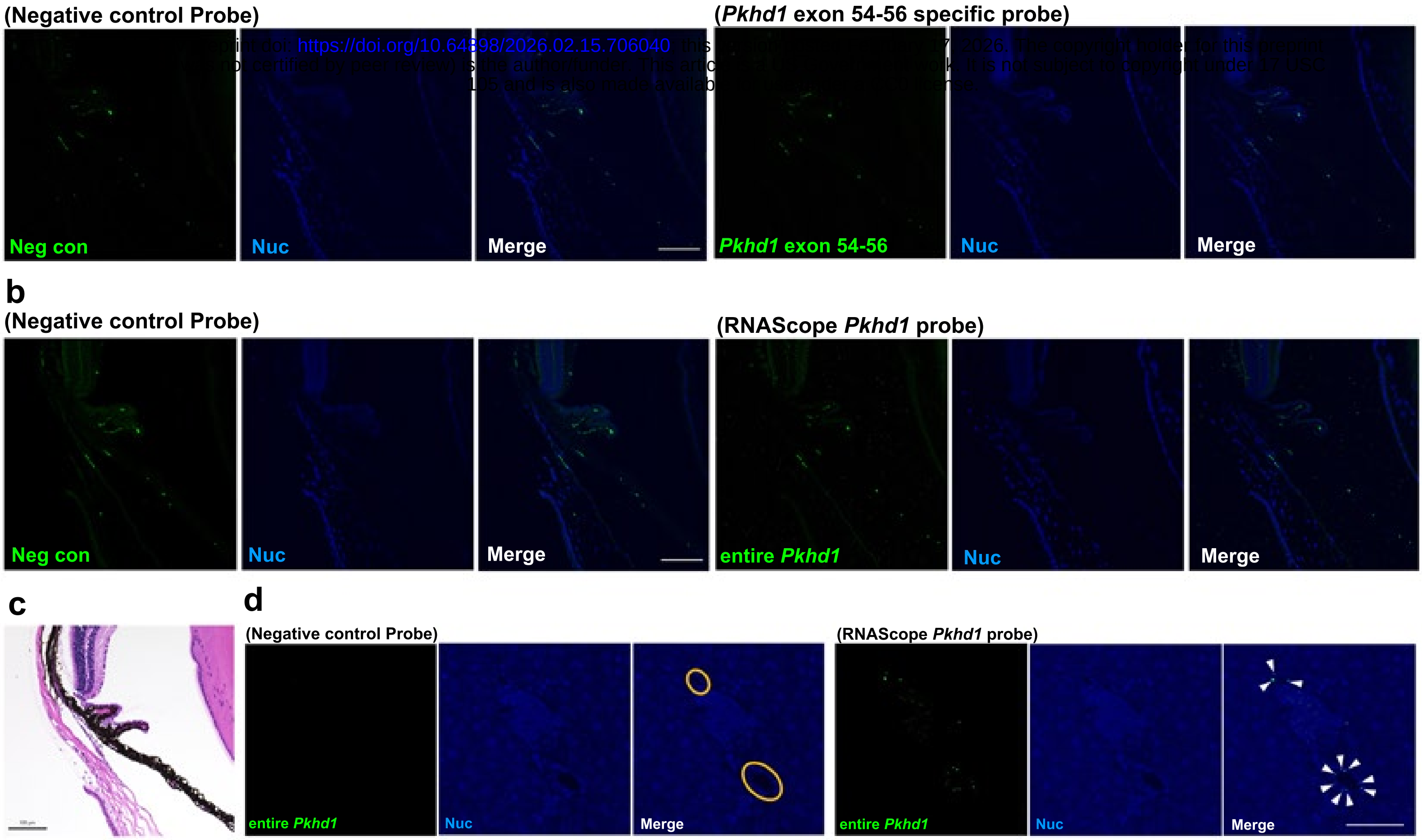

(a, b) Representative images of *in situ* hybridization of WT mouse eye at 2 months of age using RNAScope HiPlex v2. (a) Results for a negative control and a probe targeting *Pkhd1* exons 54-56 (Round 1). Scale bar 100 μm. The signal was similar in the two, consistent with a non-specific pattern. (b) Results for a negative control and a probe targeting the entire *Pkhd1* gene (Round 2). Scale bar 100 μm. No *Pkhd1*-specific signal was detected.

(c) H&E staining of the sample used for *in situ* hybridization. Scale bar 100 μm.

(d) Representative images of in situ hybridization of a wild-type mouse liver at 2 months of age using RNAScope HiPlex v2 probes for negative control and entire *Pkhd1* gene. Scale bar 50 μm. Upper: stained with negative control probe, bottom: stained with *Pkhd1* gene probe that targets the full-length transcript. Since *Pkhd1* is highly expressed in cholangiocytes, this experiment was conducted as a positive control for the eye study. Orange circles shown in the merged image of negative control probe indicate bile ducts. Arrowheads shown in the merged image of *Pkhd1* probe indicate *Pkhd1* transcripts localized to cholangiocytes. These results indicate that the *in situ* hybridization worked well but *Pkhd1* transcripts likely are below the level of detection of the assay in the eye.

Supplementary Fig.7: ZO-1 and AP-2β staining in *Pkhd1*$^{del3\text{-}67/del3\text{-}67}$ eyes.

https://doi.org/10.64898/2026.02.15.706040 ... 2026. The copyright holder for this preprint ... It is not subject to copyright under 17 USC ... use under a CC0 license.

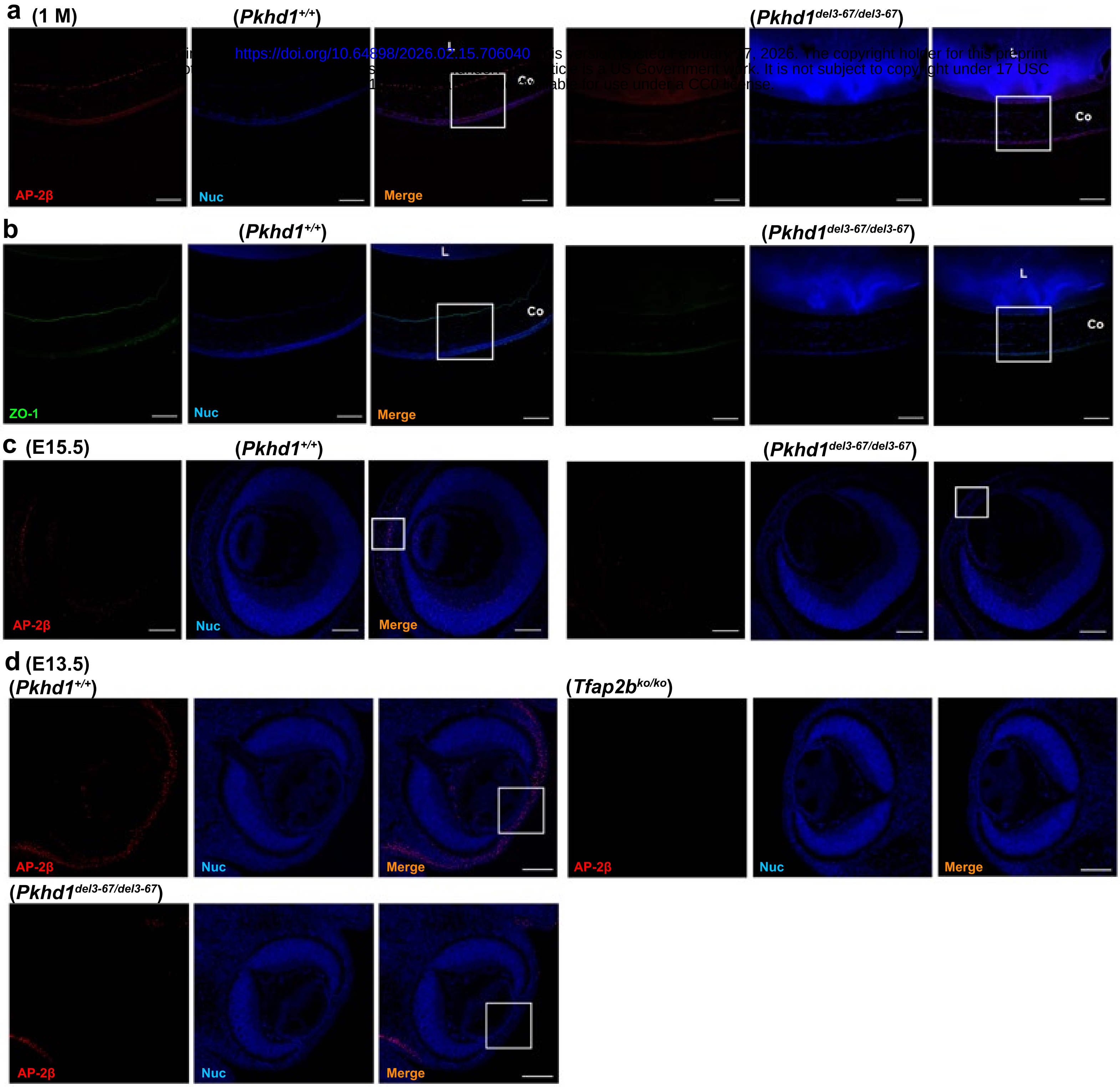

(a) Immunostaining of AP-2β in 1 month old WT and littermate *Pkhd1*$^{del3\text{-}67/del3\text{-}67}$ mouse eyes. AP-2β (red), and nuclear staining with Hoechst 33342 (blue). Scale bar 100 μm. The area highlighted in merged images is shown in Figure 3a. (b) Immunostaining of ZO-1 in specimens from the same mice in panel A. ZO-1 (green), and nuclear staining with Hoechst 33342 (blue). Scale bar 100 μm. The area highlighted in merged images is shown in Figure 3b.

(c) Immunostaining of AP-2β in E15.5 WT and *Pkhd1*$^{del3\text{-}67/del3\text{-}67}$ mouse eyes. AP-2β (red), and nuclear staining with Hoechst 33342 (blue). Scale bar 100 μm. The area highlighted in merged images is shown in Figure 3c.

(d) E13.5 control, *Tfap2b*$^{ko/ko}$ and *Pkhd1*$^{del3\text{-}67/del3\text{-}67}$ mouse eyes. AP-2β (red), and nuclear staining with Hoechst 33342 (blue). Scale bar 100 μm. The area highlighted in merged images is shown in Figure 3d. This image serves as a negative control for Figure 3d.

Supplementary Fig.8: AP-2β expression in early embryonic mouse.

bioRxiv [illegible] doi: https://doi.org/10.64898/2026.02.15.706040 [illegible]
(which w[illegible] license

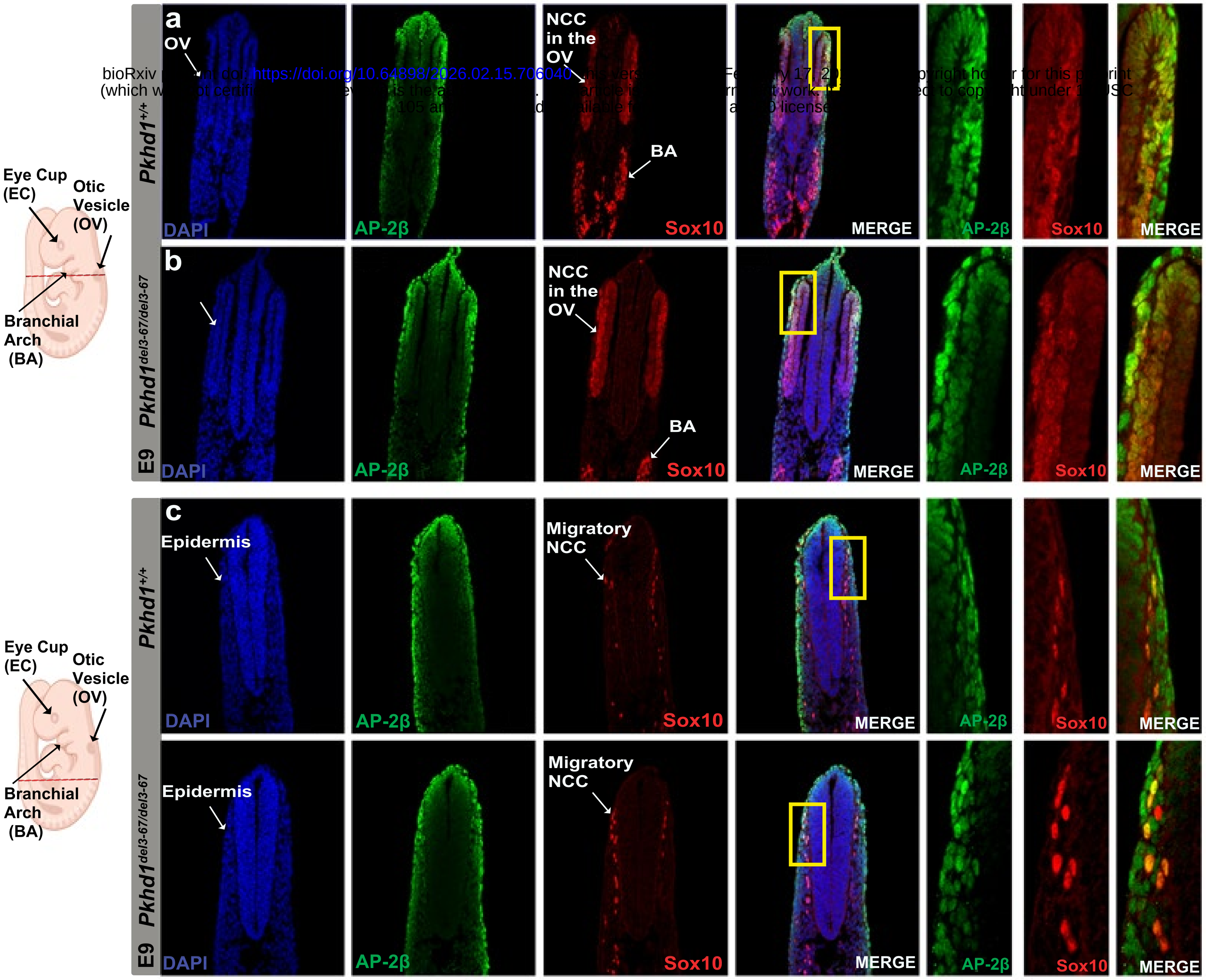

On the left are two schematic diagrams of an E9 mouse embryo depicting with a red line the respective plane of cross section presented in Supplementary Figures 8a-d.

(a, b) Cryosections from the hindbrain level showing equal levels of AP-2β (green) and Sox10 (red) expression in neural crest derived cells in the otic vesicle and branchial arches in wild type and mutant E9 embryos.

(c, d) Cryosections from the posterior axial level show a similar pattern of migrating neural cells with equal levels of AP-2β (green) and Sox10 (red) expression in wild type and mutant E9 embryos. Yellow insets show a magnification of the squared area showing co-localization of AP-2β and Sox10 in the neural crest and its derivatives. Note that AP-2β is expressed also in the epidermis.

Supplementary Fig.9: Structural variation analysis of Whole Genome Sequencing results of the *Pkhd1*$^{del3\text{-}67/del3\text{-}67}$ mouse line.

bioRxiv preprint doi: https://doi.org/10.64898/2026.02.15.706040; this version posted February 17, 2026. The copyright holder for this preprint (which [redacted] not certified by peer review) is the author/funder. This article is [redacted] work. It is not sub[redacted]opyright under 17 USC 105 and is also made available for use under a CC0 license.

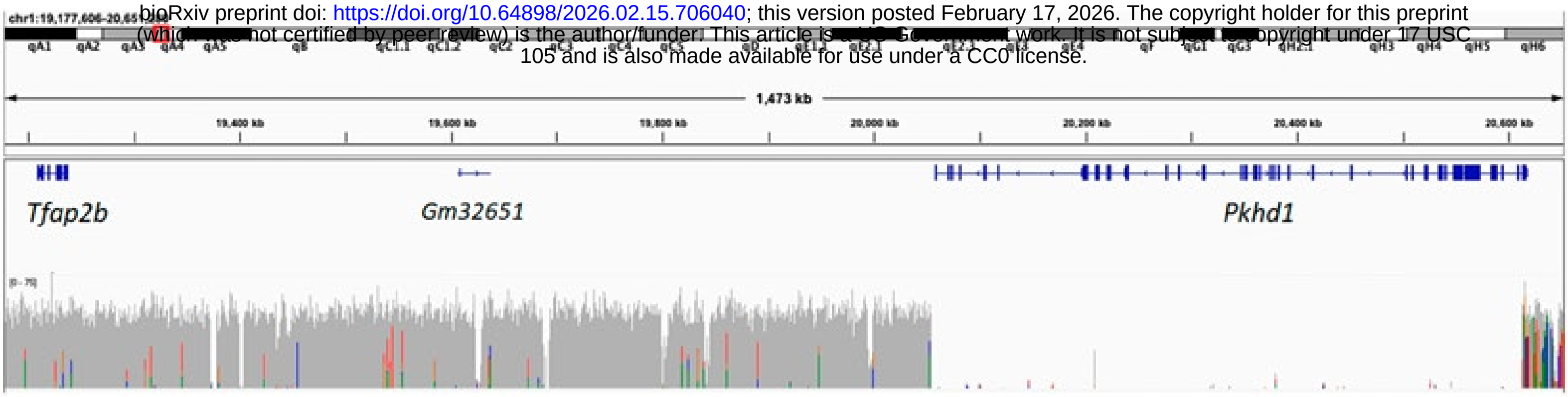

Whole genome sequence showing the *Pkhd1* and *Tfap2b* regions in a mutant *Pkhd1*$^{del3\text{-}67/del3\text{-}67}$ mouse, visualized using Integrative Genomics Viewer relative to mouse reference genome GRCm38/mm10. The vertical bars correspond to number of reads covering the region, color coded to show the proportion of reads with nucleotide mismatches (T: red; A: green; G: gold; C: blue) relative to the reference genome. Note the absence of reads in *Pkhd1* exons in the intended deleted region (chr1:20,053,820-20,613,713). Areas of low read density outside the deletion area correspond to genomic segments with high between-strain variability (Lilue, J., et al.,

D1243-D1249) or blacklisted as problematic sequencing regions (Amemiya, H., et al. *Sci Rep*, 2019. 9(1): p. 9354).

Supplementary Fig.10: Additional data for *Tfap2b*$^{ko/ko}$ mouse.

bioRxiv preprint doi: https://doi.org/10.64898/2026.02.15.706040; this version posted February 17, 2026. The copyright holder for this preprint (which was not certified by peer review) is the author/funder. This article is a US Government work. It is not subject to copyright under 17 USC 105 and is also made available for use under a CC0 license.

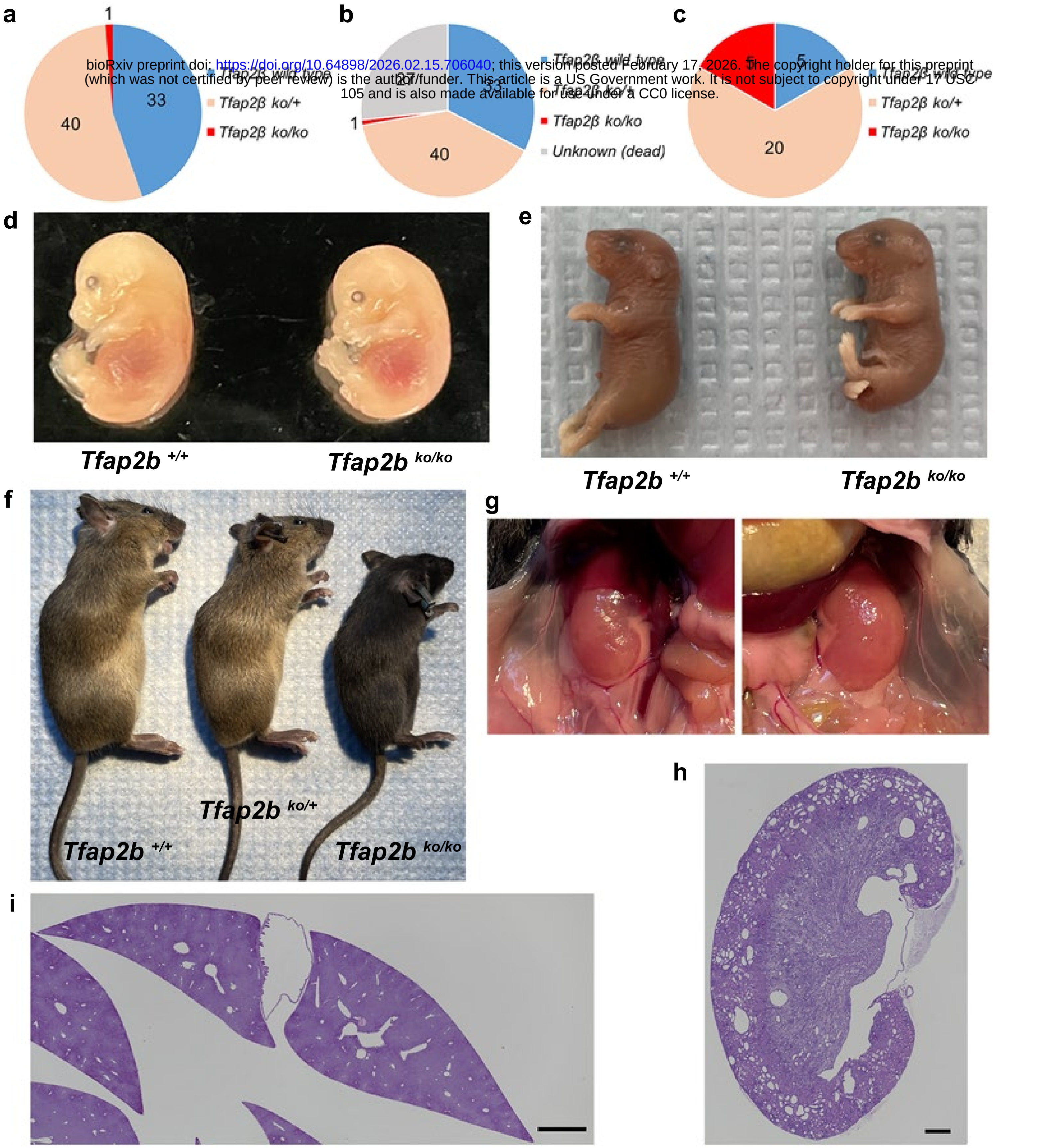

(a) Genotyping results of weaned pups from *Tfap2b*$^{ko/+}$ x *Tfap2b* $^{ko/+}$ mating. Genotypes were determined for 74 pups weaned from 15 litters at 3 weeks of age. Only 1 *Tfap2b*$^{ko/ko}$ survived until weaning.

(b) Genotyping results of delivered pups from *Tfap2b*$^{ko/+}$ x *Tfap2b* $^{ko/+}$ mating. A total of 101 pups from 15 litters were delivered but 27 died before weaning and their genotypes could not be determined. Same cohort used in Supplementary Figure 10a.

(c) Genotyping results of E 15.5 mouse embryos from *Tfap2b*$^{ko/+}$ x *Tfap2b* $^{ko/+}$ mating. A total of 20 embryos from three litters were evaluated. The number of *Tfap2b* $^{+/+}$ and *Tfap2b*$^{ko/ko}$ embryos was the same at this age suggesting no early embryonic lethality in *Tfap2b*$^{ko/ko}$.

(d) Images of E13.5 *Tfap2b*$^{+/+}$ and *Tfap2b*$^{ko/ko}$ mouse embryo littermates.

(e) Images of E18.5 *Tfap2b*$^{+/+}$ and *Tfap2b*$^{ko/ko}$ mouse embryo of littermates.

(f) Images of P40 *Tfap2b*$^{+/+}$, *Tfap2b*$^{ko/+}$ and *Tfap2b*$^{ko/ko}$ littermates. All mice are female. The sole surviving *Tfap2b*$^{ko/ko}$ was smaller than her littermates.

(g) Images of the cystic kidneys of P40 *Tfap2b*$^{ko/ko}$ mouse.

(h) Representative H&E staining of cystic kidney of *Tfap2b*$^{ko/ko}$. Scale bar 500 μm.

(i) Representative H&E staining of the liver of *Tfap2b*$^{ko/ko}$. Scale bar 1000 μm. No cystic changes were observed in the liver of *Tfap2b*$^{ko/ko}$.

Supplementary Fig.11. ***Tfap2b**$^{ko/ko}$* **mouse eye phenotype.**

bioRxiv preprint doi: https://doi.org/10.64898/2026.02.15.706040; this version posted February 17, 2026. The copyright holder for this preprint (which was not certified by peer review) is the author/funder. This article is a US Government work. It is not subject to copyright under 17 USC 105 and is also made available for use under a CC0 license.

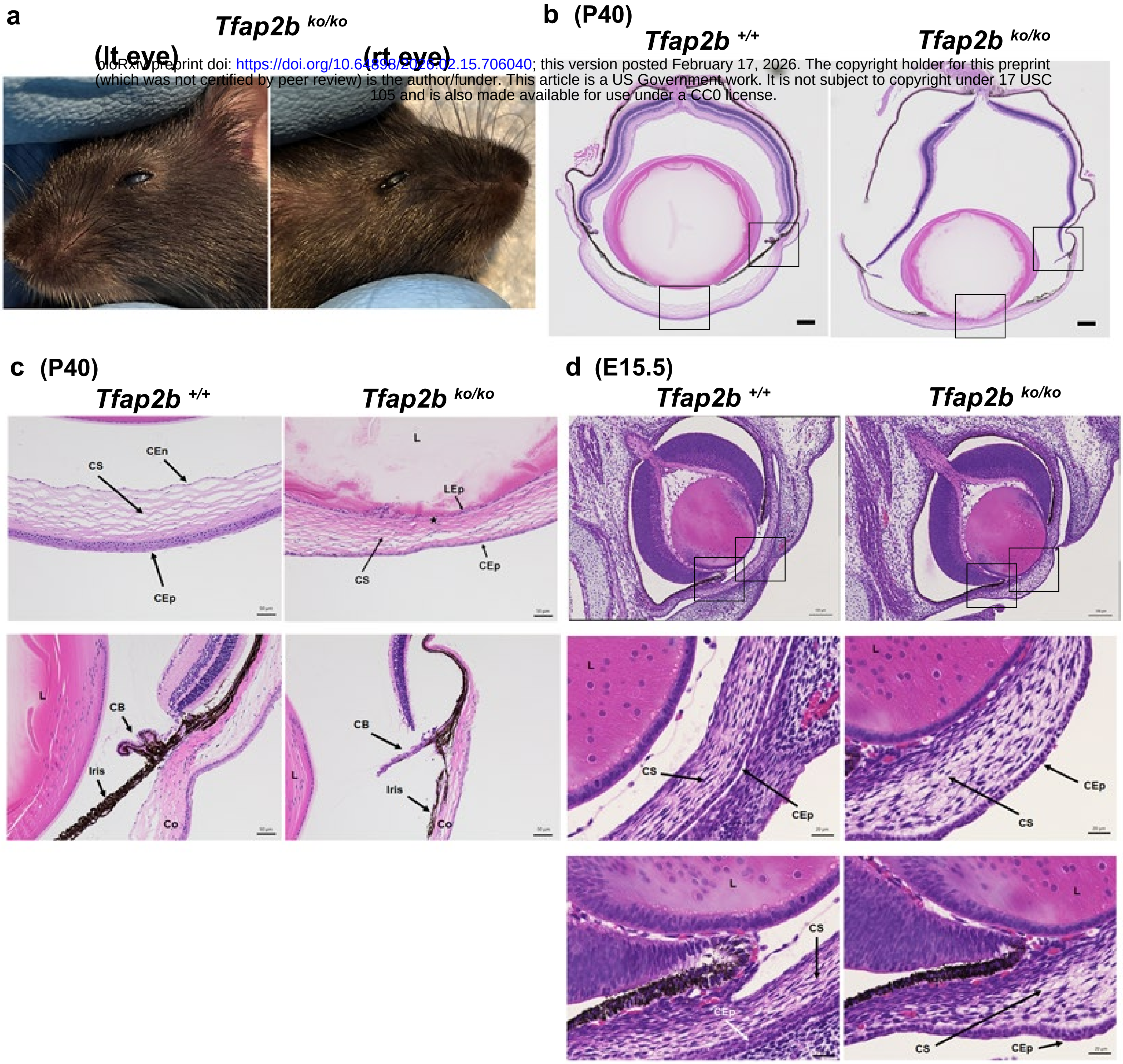

(a) Image of the eyes of *Tfap2b*$^{ko/ko}$ at P40. The *Tfap2b*$^{ko/ko}$ mouse had developed corneal opacity in both eyes.

(b) Representative eye pathology with H&E staining at P40. Whole eye images of P40 *Tfap2b*$^{+/+}$ and *Tfap2b*$^{ko/ko}$ littermates are shown. Black squares indicate the region shown in Supplementary Figure 11c. Scale bar 200 μm.

(c) Representative eye pathology with H&E staining of P40 *Tfap2b*$^{+/+}$ and *Tfap2b*$^{ko/ko}$ littermates. Upper: cornea with 50 μm scale bar, bottom: angle tissue with 50 μm scale bar. CB: ciliary body, Co: cornea, CEn: corneal endothelium, CS: corneal stroma, CEp: corneal epithelium, L: lens, LEp: lens epithelium. star indicates absent corneal endothelium.

(d) Representative eye pathology with H&E staining of E15.5 *Tfap2b*$^{+/+}$ and *Tfap2b*$^{ko/ko}$ littermate. Top: whole eye image with 100 μm scale bar. Black squares indicate the region of cornea and angle tissue. Middle: cornea with 20 μm scale bar, bottom: angle tissue with 20 μm scale bar. CS: corneal stroma, CEp: corneal epithelium, L: lens.

Supplementary Fig.12: Additional data for *Pkhd1$^{del3-67+}$;Tfap2b$^{ko/+}$* trans-heterozygote mouse phenotype.

bioRxiv preprint doi: https://doi.org/10.64898/2026.02.15.706040; this version posted February 17, 2026. The copyright holder for this preprint (which was not certified by peer review) is the author/funder. This article is a US Government work. It is not subject to copyright under 17 USC 105 and is also made available for use under a CC0 license.

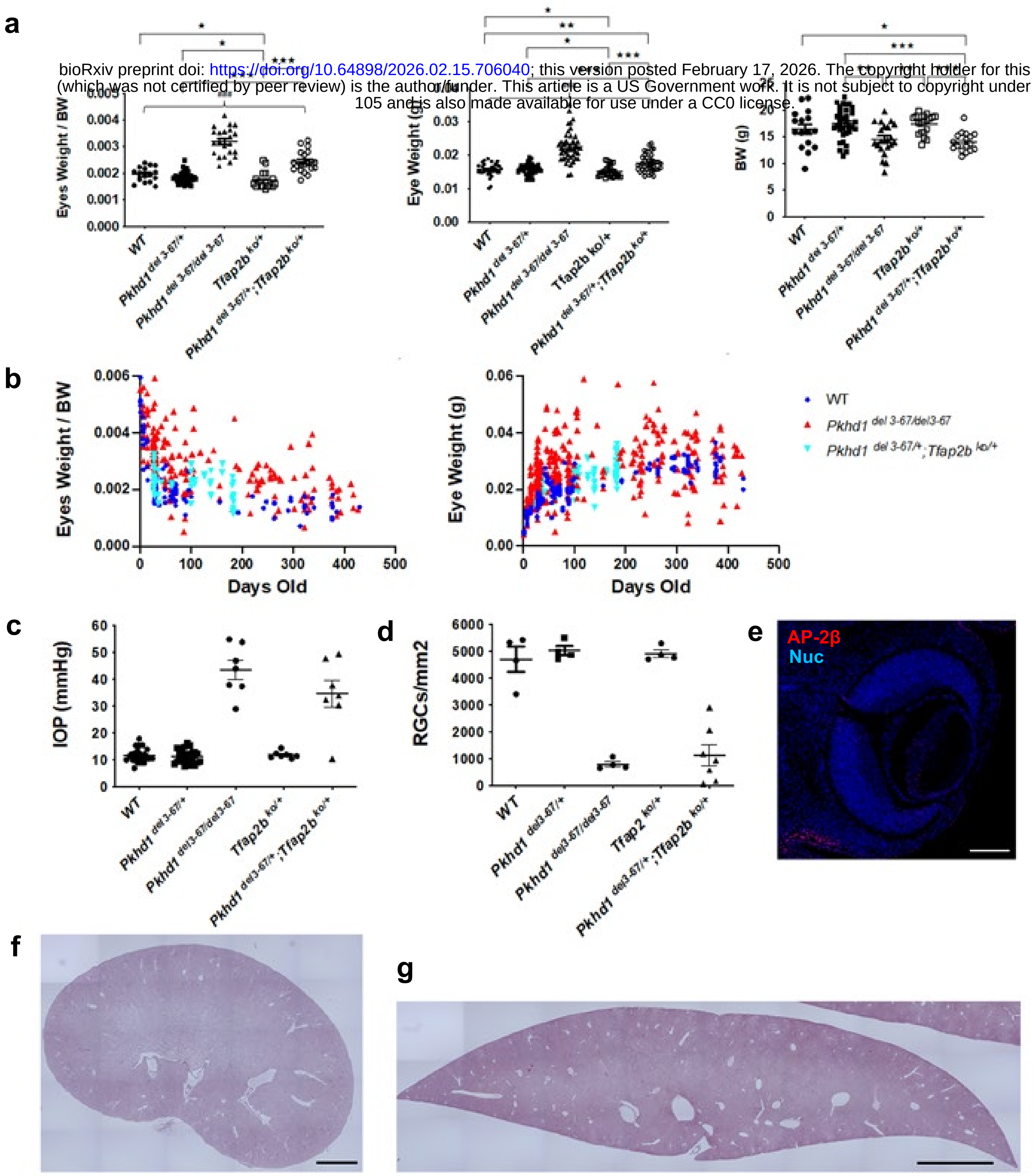

(a) From left: Eyes weight per body weight (BW), eye weight and BW at 4 weeks of age including all mouse genotypes. A subset of these data is also presented in Figure 6e. N=16 mice (9 males and 7 females), *Pkhd1$^{del3-67+}$* N=33 mice (15 males and 18 females), *Pkhd1$^{del3-67del3-67}$* N=32 mice (16 males and 16 females), *Tfap2b$^{ko/+}$* N=20 mice (16 males and 4 females), trans-heterozygote N=18 mice (7 males and 11 females). For Supplementary Figure 12 a and b, each dot in Eyes Weight/BW is the mean value of eyes from a single mouse while each dot in eye weight indicates the measurement for a single eye. Only significant differences are shown: *** $p < 0.001$, ** $p < 0.01$. * $P < 0.05$.

(b) Scatter plot of eyes weight per BW and eye weights of WT, *Pkhd1$^{del3-67/del3-67}$* and *Pkhd1$^{del3-67+}$;Tfap2b$^{ko/+}$* trans-heterozygote at different ages. Other genotypes were not included to improve clarity. Left: scatter plots of eyes weight per BW. Right; eye weight (in grams).

(c) IOP of all mouse genotypes at 3 weeks of age. A subset of these data is also presented in Figure 6f. Each dot is the mean value of IOP for a single mouse.

(d) Number of RGC at 4 weeks of age from all mouse genotypes. A subset of these data is also presented in Figure 6g. Each dot represents the mean number of RGC counted from a series of immunostained images from a single mouse.

(e) An E13.5 *Pkhd1$^{del3-67+}$;Tfap2b$^{ko/+}$* trans-heterozygote mouse eye stained for AP-2β showing absent staining in most structures but some positive cells in the POM, as had been seen with the E13.5 *Pkhd1$^{del3-67/del3-67}$* specimens. This example illustrates the similar patterns of AP-2β staining observed in *Pkhd1$^{del3-67/del3-67}$* and *Pkhd1$^{del3-67+}$;Tfap2b$^{ko/+}$* trans-heterozygous samples. Five of the *Pkhd1$^{del3-67+}$;Tfap2b$^{ko/+}$* trans-heterozygous eyes had the pattern shown in Figure 6l while one had the pattern shown here. Conversely, one E13.5 *Pkhd1$^{del3-67del3-67}$* specimen lacked staining in the POM as shown in Figure 6f for the *Pkhd1$^{del3-67+}$;Tfap2b$^{ko/+}$* trans-heterozygote. AP-2β (red), and nuclear staining with Hoechst 33342 (blue). Scale bar 100 μm.

(f) Representative H&E image of a non-cystic kidney of a *Pkhd1$^{del3-67+}$;Tfap2b$^{ko/+}$* trans-heterozygote. Scale bar 1000 μm.

(g) Representative H&E image of the non-cystic liver of a *Pkhd1$^{del3-67+}$;Tfap2b$^{ko/+}$* trans-heterozygote. Scale bar 2000 μm.

Supplementary Fig.13: Incisor malocclusion phenotype.

bioRxiv preprint doi: https://doi.org/10.64898/2026.02.15.706040; this version posted February 17, 2026. The copyright holder for this preprint (which was not certified by peer review) is the author/funder. This article is a US Government work. It is not subject to copyright under 17 USC 105 and is also made available for use under a CC0 license.

a ***Pkhd1*** $^{del3\text{-}67/+}$**/*Tfap2b*** $^{ko/+}$

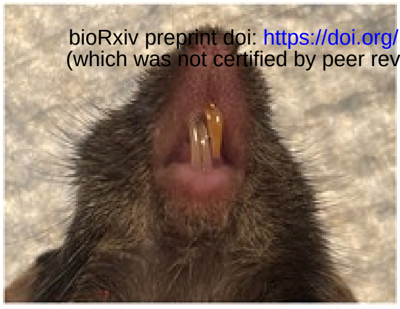

b

Genotype	Number of mice	Diagnosed as Malocclusion
WT	23	0/23 = 0 %
Pkhd1 $^{del3\text{-}67/+}$	34	0/34 = 0 %
Tfap2b $^{ko/+}$	23	0/23 = 0 %
Pkhd1 $^{del3\text{-}67/+}$ / *Tfap2b* $^{ko/+}$	25	22/25 = 88 %

c ***Pkhd1*** $^{del3\text{-}67/del3\text{-}67}$

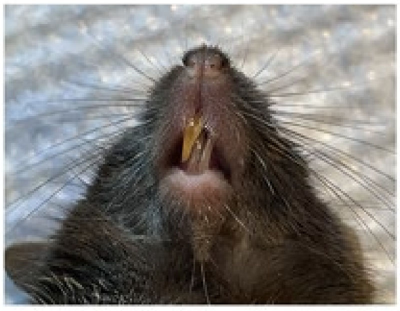

d

Pkhd1 Genotype	Number of mice	Diagnosed as Malocclusion
WT	203	0/203 = 0 %
Pkhd1 $^{del3\text{-}67/+}$	484	0/484 = 0 %
Pkhd1 $^{del3\text{-}67/del3\text{-}67}$	178	171/178 = 96.1 %

e ***Tfap2b*** $^{ko/ko}$

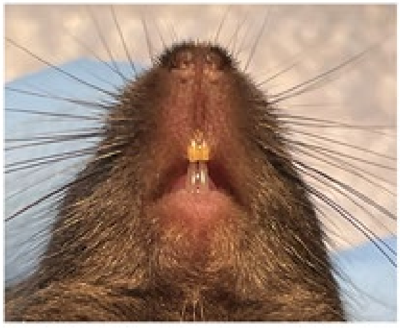

(a) Representative image of incisor malocclusion of a *Pkhd1* $^{del3\text{-}67+}$;*Tfap2b* $^{ko/+}$ trans-heterozygous mouse.

(b) Prevalence of malocclusion in pups related to the *Pkhd1* $^{del3\text{-}67+}$;*Tfap2b* $^{ko/+}$ trans-heterozygous mouse line. Data include pups from *Pkhd1* $^{del3\text{-}67/del3\text{-}67}$ x *Tfap2b* $^{ko/+}$ mating in addition to the pups shown in Figure 6d. All mice that did not develop malocclusion developed eye phenotype (data not shown).

(c) Representative image of incisor malocclusion of *Pkhd1* $^{del3\text{-}67/del3\text{-}67}$ mouse.

(d) Prevalence of malocclusion in the *Pkhd1* $^{del3\text{-}67}$mouse line.

(e) Image of incisor of *Tfap2b* $^{ko/ko}$ mouse shown in Supplementary Figure 11a.
